# Suppressive effects of iron overloading on vascular calcification in uremic rats

**DOI:** 10.1007/s40620-014-0046-3

**Published:** 2014-02-06

**Authors:** Takuya Seto, Chieko Hamada, Yasuhiko Tomino

**Affiliations:** Juntendo University School of Medicine, Bunkyo-ku, Tokyo, Japan

**Keywords:** Iron dextran, Osteoblastic transdifferentiation, Mönckeberg medial calcification, Runx2, Pit-1

## Abstract

**Background:**

Medial vascular calcification is a specific complication in chronic kidney disease (CKD) patients although its pathogenesis is poorly understood. The administration of iron (Fe), generally used for the treatment of anemia in CKD patients, induces oxidative stress. Fe loading possibly affects the progress of vascular calcification in uremia. We investigated the effect of Fe on vascular calcification and its mechanism in uremic rats.

**Method:**

Thirty-two rats were divided into four groups: untreated rats (controls), rats fed a standard diet with Fe administration (Fe group), rats fed an adenine-enriched diet (uremic group), and rats fed an adenine-enriched diet with Fe administration (uremic + Fe group). Iron dextran was administered once a week for 5 weeks intraperitoneally. Morphological alterations and vascular calcification-associated factors in the aortic wall were evaluated.

**Results:**

No aortic calcification was observed in the control group although uremic rats developed severe vascular calcification. Fe loading suppressed vascular calcification in the uremic groups. Expressions of runt-related transcription factor 2 (Runx2), single-strand (ss)DNA and phosphate transporter (Pit)-1 were increased in the uremic rats compared to the control rats. In the uremic group, Fe administration did not show any effect on ssDNA expression, but reduced Runx2 and Pit-1 expressions.

**Conclusion:**

Fe suppressed the development of vascular calcification through the prevention of Pit-1 and vascular smooth muscle cell osteoblastic transdifferentiation.

## Introduction

Chronic kidney disease (CKD) is an independent risk factor for the development of cardiovascular disease (CVD) and the increased morbidity and mortality associated with CVD [1]. Medial calcification is a characteristic finding in vascular pathological change in end-stage kidney disease [2]. This pathologic mechanism is caused by a metabolic abnormality of calcium and phosphate [3]. Previous studies have revealed that hyperphosphatemia caused by CKD triggers the mechanism for transformation of vascular smooth muscle cells (VSMCs) to chondrocytes or osteoblast-like cells, and medial-based calcification deposits [4].

Endothelial dysfunction corresponding to a uremic condition plays an important role in the development of CVD. Oxidative stress is generally known as one of the factors affecting endothelial dysfunction and the development of atherosclerosis [5]. Iron (Fe) loading increases oxidative stress and causes endothelial dysfunction. While ferritin preserves Fe excess, it counteracts the toxicity of Fe and protects against oxidative stress [6]. However, there has been no investigation into the effect of Fe administration on vascular calcification in the uremic condition in vivo. The objective of the present study was to evaluate the effect of Fe on the development of vascular calcification and its mechanism in uremic rats.

## Materials and method

### Laboratory animals and experimental design

Experiments were performed on 32 Sprague–Dawley male rats at 10 weeks of age purchased from Charles River Japan (Kanagawa Prefecture, Japan). The study protocol was reviewed and approved by the Animal Care and Use Committee of the Juntendo University Faculty of Medicine. Before the start of this study, the rats were conditioned on a standard diet (CE-2; 1.02 % phosphorus, 1.08 % calcium, 25.1 % crude protein and 2.4 IU/g vitamin D_3_) (CLEA Japan, Tokyo, Japan) for 2 weeks. The rats were housed in cages (two or three animals in each cage) and allowed free access to food and tap water. The animal room was kept on a 12 h light/dark cycle (7:00 a.m. to 7:00 p.m. in the dark, 7:00 p.m. to 7:00 a.m. in light), at a constant temperature (22 ± 1 °C) and relative humidity of 55 ± 5 % throughout the experimental period.

**Fig. 1:**
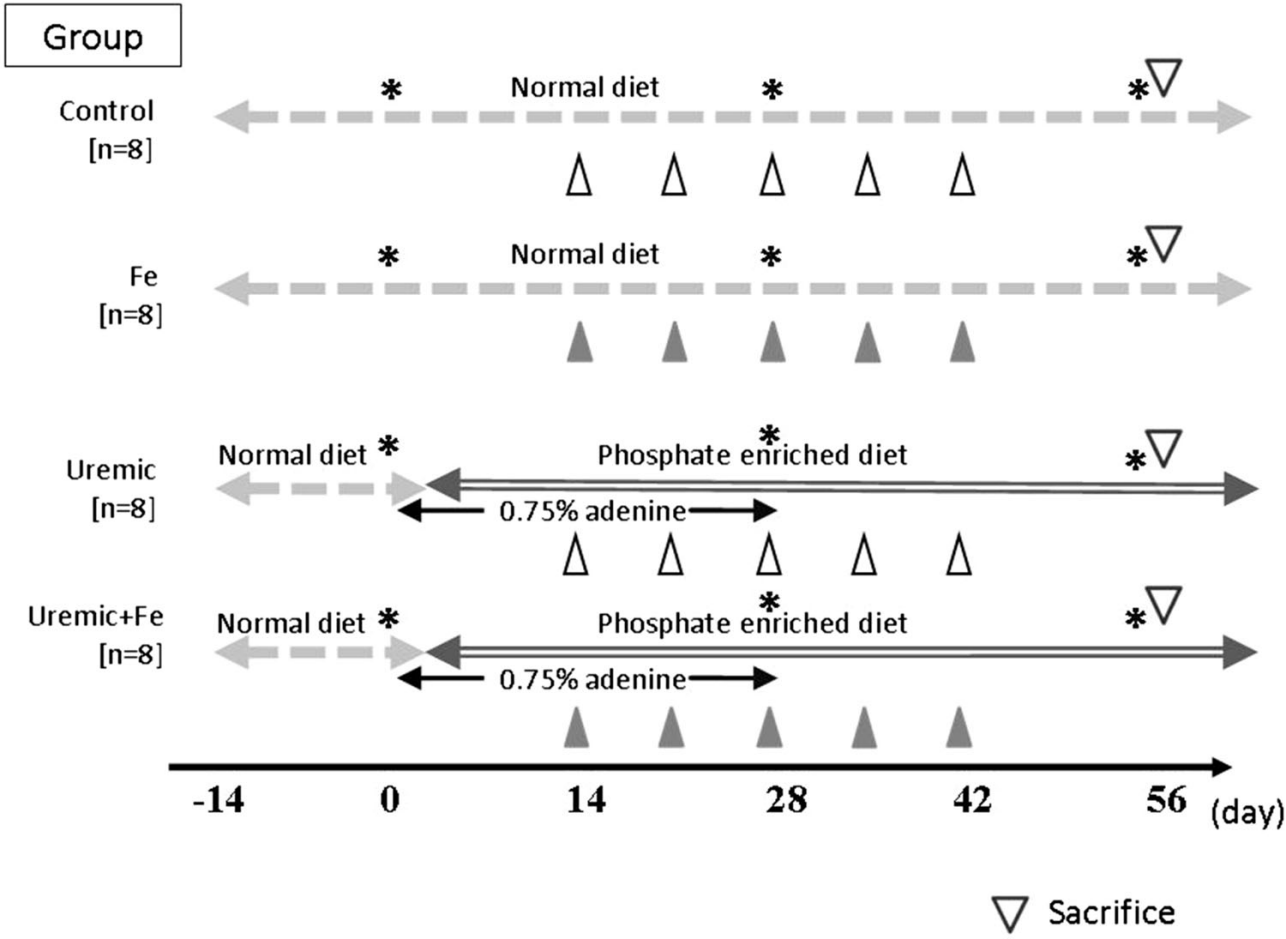
Experimental design. Body weight and blood pressure were measured, and blood samples were taken on days 0, 28 and 56. *Closed triangles* indicate administration of 40 mg iron dextran (Fe) intraperitoneally. *Open triangles* indicate administration of 0.4 ml sterile saline intraperitoneally. *Stars* indicate the times of blood sampling and measuring blood pressure and body weight

After the conditioning period, the 32 rats were divided into two groups, i.e. normal (standard diet) and adenine diet. Sixteen rats were fed a 0.75 % adenine-containing (Sigma Chemical Co., St Louis, MO, USA), phosphate-enriched diet (1.20 % phosphorus, 1.12 % calcium, 25.1 % crude protein and 2.4 IU/g vitamin D_3_) (CLEA Japan, Tokyo, Japan) to induce uremia for 4 weeks [7]. In the 4 weeks following adenine withdrawal, the rats were fed a simple phosphate-enriched diet until sacrifice (uremic group). The other 16 rats were continuously fed the standard diet for 8 weeks until sacrifice (control group). The experimental design is summarized in Fig. 1.

### Administration of iron dextran (Fe)

We injected 40 mg of Fe (100 g Fe^2+^/l; Sigma, St Louis, MO, USA) intraperitoneally on days 14, 21, 28, 35 and 42 into 8 normal diet rats and 8 adenine diet rats. Sterile saline at 0.4 ml was injected as a control in the other 8 normal diet rats and other 8 adenine diet rats. Accordingly, the 32 rats were divided into four groups of eight animals each, as follows: the control group, the Fe only group, the uremic group and the uremic + Fe group. On day 56, all rats were sacrificed by heart puncture under ether anesthesia according to the guidelines of the Animal Care and Use Committee of the Juntendo University Faculty of Medicine.

### Body weight and blood pressure

Body weight and blood pressure were measured on days 0, 28 and 56. Systolic arterial blood pressure (SBP) of pre-warmed rats was measured by the tail-cuff method (BP-98A; Softron, Tokyo, Japan).

### Blood sampling and analysis

Blood samples were obtained on days 0, 28 and 56 in each group from the tail vein and were also obtained by heart puncture under ether anesthesia on day 56. Serum creatinine (Cr), hematocrit (Ht), iron (Fe), transferrin saturation (TS), ferritin, corrected calcium, phosphorus, intact parathyroid hormone, 25(OH)D_3_, 1,25(OH)_2_D_3_, fibroblast growth factor 23 (FGF23), alanine aminotransferase (ALT), aspartate aminotransferase (AST) and alkaline phosphatase (ALP) were measured in a commercial laboratory (SRL Co. Tachikawa City, Japan).TS was calculated from the ratio of serum Fe: total iron-binding capacity.

### Quantitative assessments of vascular calcification

The thoracic aorta was fixed in neutral buffered formalin and then cut into 2–3 mm thick rings that were embedded upright in the same paraffin block. Every section comprised an average of 12 cross-sections of the aorta at different sites along the vessel. Sections of 4 μm were stained using the von Kossa method. Vascular calcification was then evaluated histomorphometrically with image analysis software, Image-Pro4.5J (Planetron, Tokyo, Japan), at 100× magnification. Absolute whole vascular walls and the calcified areas were measured for each animal, and the calcification ratio was expressed as the percentile of calcification area in vascular wall by the following formula:

Calcification ratio (%) = von kossa − positive area (μm^2^)/total vascular wall area (μm^2^) × 100.

### Immunohistochemistry

Thoracic aorta were fixed in 10 % phosphate-buffered formalin and embedded in paraffin. The paraffin-embedded specimens were sliced into 5-in. sections and stored at room temperature prior to use. Routine histology (hematoxylin and eosin staining) was performed in order to evaluate the basic histomorphological features of the specimens. All sections were deparaffinized in xylene, followed by 100 % ethanol, and then placed in a freshly prepared methanol/0.3 % H_2_O_2_ solution for 15 min for blocking of endogenous peroxidase activity. After washing three times with phosphate buffered saline (PBS), microwave antigen retrieval was performed with a hot 0.01 mol/l-citrate buffer (pH 6.0) for 20 min. The sections were allowed to reach room temperature before subsequent procedures were performed. The sections were incubated with a blocking solution (2 % bovine serum albumin, 2 % fetal calf serum, 0.2 % fish gelatin) at room temperature for 30 min after washing three times with PBS. The sections were then incubated with a monoclonal mouse anti runt-related transcription factor 2 (Runx2) antiserum, (Abnova Corporation, Taiwan) (1:333) or polyclonal rabbit single-strand (ss)-DNA antibody (DAKO, Carpinteria, CA, USA) (1:2,000) diluted with blocking solution at 4 °C overnight. Negative staining was confirmed by incubation without primary or secondary antiserum. These sections were incubated with the diluted peroxidase-labeled secondary antiserum (DAKO, Carpinteria, CA, USA) at room temperature for 30 min. A 3,3′-diaminobenzidine chromogen substrate solution was applied for 2 min to develop the stain. The sections were counterstained with hematoxylin, mounted, and then coverslipped until microscopic analyses. The number of Runx2 or ss-DNA positive cells in vascular walls was separately counted in each specimen (1,400 μm × 1,000 μm) for quantitative assessments. The cell density in each area was calculated as the cell count divided by the total area by examiners blinded to the treatment regimen.

### Quantitative real-time reverse transcriptase-polymerase chain reaction (PCR) analysis

Total RNA was extracted from the frozen thoracic aorta using a trizol reagent (Invitrogen AG, Basel, Switzerland) and the RNeasy Mini Kit (Qiagen K.K., Tokyo, Japan). A 16 μl reaction mixture containing 1 μg of RNA, 4 μl of 2.5 mmol/l dNTP mixture (Takara Biochemicals, Ohtsu City, Japan) and 2 μl of Random Decamers RETROscript (Ambion Inc., Austin, TX, USA) in RNase-free water was inactivated by heating at 70 °C for 3 min. The product was added to 2 μl of a 10× PCR buffer (Takara Bio Inc., Shiga, Japan), a 1 μl of Protector RNase Inhibitor (Roche Diagnostics Corp., Mannheim, Germany) and 0.5 μl of Moloney murine leukemia virus reverse transcriptase (Invitrogen Corp., Carlsbad, CA, USA), followed by incubation at 42 °C for 60 min. A 2 μl aliquot of diluted complementary (c)DNA, 1.6 μl forward primers, 1.6 μl reverse primer, 10 μl SYBR Green PCR Master Mix (Applied Biosystems, Carlsbad, CA, USA) and 4.8 μl of cDNA-free double-distilled water were then mixed to obtain a final reaction mixture of 20 μl according to the manufacturer’s instructions. The mixture was denatured and amplified using the 7500 Real-Time PCR system (Applied Biosystems) under the following conditions: (i) 20 s at 95 °C for 1 cycle, (ii) 3 s at 95 °C and 30 s at 60 °C for 40 cycles, (iii) 15 s at 95 °C, 60 s at 60 °C, 15 s at 95 °C and 15 s at 60 °C for 1 cycle. cDNA-free double-distilled water as negative control was included in each reaction. For quantification of the PCR product, the samples were standardized with a PCR product for glyceraldehydes-3-phosphate dehydrogenase. The PCR primers were designed as follows: Runx2, 5-ACAGAACCACAAGTGCGGTGCAA-3′ (forward),5′-TGGTCTCGGTGGCTGGTAGTGA-3′ (reverse) and phosphate transporter (Pit)-1, 5′-TACCATCCTCATCTCGGTGG-3 (forward), 5′-TGACGGCTTGACTGAACTGG-3 (reverse).

### Statistical analysis

All numerical data are expressed as mean ± SD. Differences between the groups were examined for statistical significance by analysis of variance. A p value <0.05 was considered as a statistically significant difference. Statistical analyses were performed using Stat View 5.0 software (Abacus Concepts, Inc., Stanford, CA, USA).

## Results

### Biochemical characteristics and biochemical findings among the groups

The biochemical characteristics and biochemical findings of the four groups are shown in Table 1. Adenine feeding for 4 weeks induced a ten-fold elevation of serum creatinine in rats compared to normal diet. Uremic condition induced by adenine-feeding decreased the levels of hematocrit. Fe loading did not improve the renal function and anemia in the uremic + Fe group. Levels of serum iron concentration (Fe), transferrin saturation (TS) and serum ferritin in Fe-treated rats (the Fe alone group and the uremic + Fe group) were significantly elevated by Fe administration. Levels of TS and serum ferritin in the uremic + Fe group were higher than those in the uremic group. Uremic condition suppressed vitamin D activation through an increase of FGF23. Fe administration reversed this phenomenon without serum phosphate deletion. In the uremic and uremic + Fe groups, serum calcium concentrations were significantly lower, and serum phosphate concentrations were higher than those in the control and Fe only groups. The level of serum phosphate did not differ between the uremic and the uremic + Fe groups. Fe administration slightly increased ALT and AST levels but did not induce liver injury. Serum ALP was increased in the uremic and the uremic + Fe groups.

**Table I:** Characteristics and biochemical parameters in treated groups on day 56

Parameters	Control group (n = 8)	Control + Fe group (n = 8)	Uremic group (n = 6)	Uremic + Fe group (n = 6)
SBP (mmHg)	121.9 ± 3.7	126.3 ± 10.8	131.7 ± 204	116.1 ± 9.3
BW (g)	534.4 ± 21.9	503.9 ± 20.7	294.5 ± 29.9^a^	296.8 ± 33.4^a,b^
Cr (mg/dl)	0.33 ± 0.03	0.34 ± 0.03	3.36 ± 0.74^a^	2.96 ± 0.84^a,b^
Ht (%)	53.9 ± 2.4	52.3 ± 3.1	27.9 ± 6.3^a^	25.7 ± 3.3^a,b^
Fe (μg/dl)	181.8 ± 36.6	323.1 ± 95.9^a^	146.0 ± 32.0	291.2 ± 47.8^a,b^
TS (%)	27.5 ± 6.2	49.2 ± 12.7^a^	40.2 ± 4.60	82.2 ± 14.1^b,c^
Ferritin (ng/ml)	2,618.8 ± 496.6	9,883.5 ± 3,757.5^a^	1,753.3 ± 491.4	15,116.7 ± 6,724.1^b,c^
Corrected Ca (mg/dl)	11.6 ± 0.37	12.3 ± 0.46	8.73 ± 1.53^a^	8.97 ± 1.15^a,b^
Pi (mg/dl)	9.03 ± 0.56	8.95 ± 0.59	17.35 ± 3.06^a^	15.28 ± 3.54^a,b^
ALT (mg/dl)	70.5 ± 29.5	138.63 ± 98.1	52.5 ± 24.5	63 67 ± 60.8
AST (mg/dl)	116.6 ± 31.6	204.5 ± 145.7	62.3 ± 11.4^a^	91.5 ± 66.3^a,b^
Intact PTH (pg/ml)	6,111.5 ± 1,578.3	425.1 ± 385.2	24,533.3 ± 9,575.7^a,b^	21,790.0 ± 10,449.0^a,b^
25(OH)D_3_ (ng/ml)	32.6 ± 5.15	32.0 ± 9.4	31.8 ± 13.0	33.6 ± 14.7
l,25(OH)_2_D_3_ (pg/ml)	133.0 ± 20.1	133.2 ± 27.1	24.0 ± 8.1^a,b^	99.8 ± 107.1^c^
FGF23 (pg/ml)	56.8 ± 12.2	103.1 ± 22.6	74,502.0 ± 10,046.2^a,b^	2,672 4 ± 3,394.8^c^

*SBP* systolic blood pressure, *BW* body weight Cr serum creatinine, *Ht* hematocrit, *Fe* serum iron, *TS* transferrin saturation, *Ca* calcium, *Pi* inorganic phosphate, *ALT* alanine aminotransferase, *AST* aspartate aminotransferase

^a^ p < 0.05 vs. the control group, ^b^ p < 0.05 vs. the control + Fe group, ^c^ p < 0.05 vs. the uremic group All data are given as mean ± SD

### Vascular medial calcification in the thoracic aorta

**Fig. 2:**
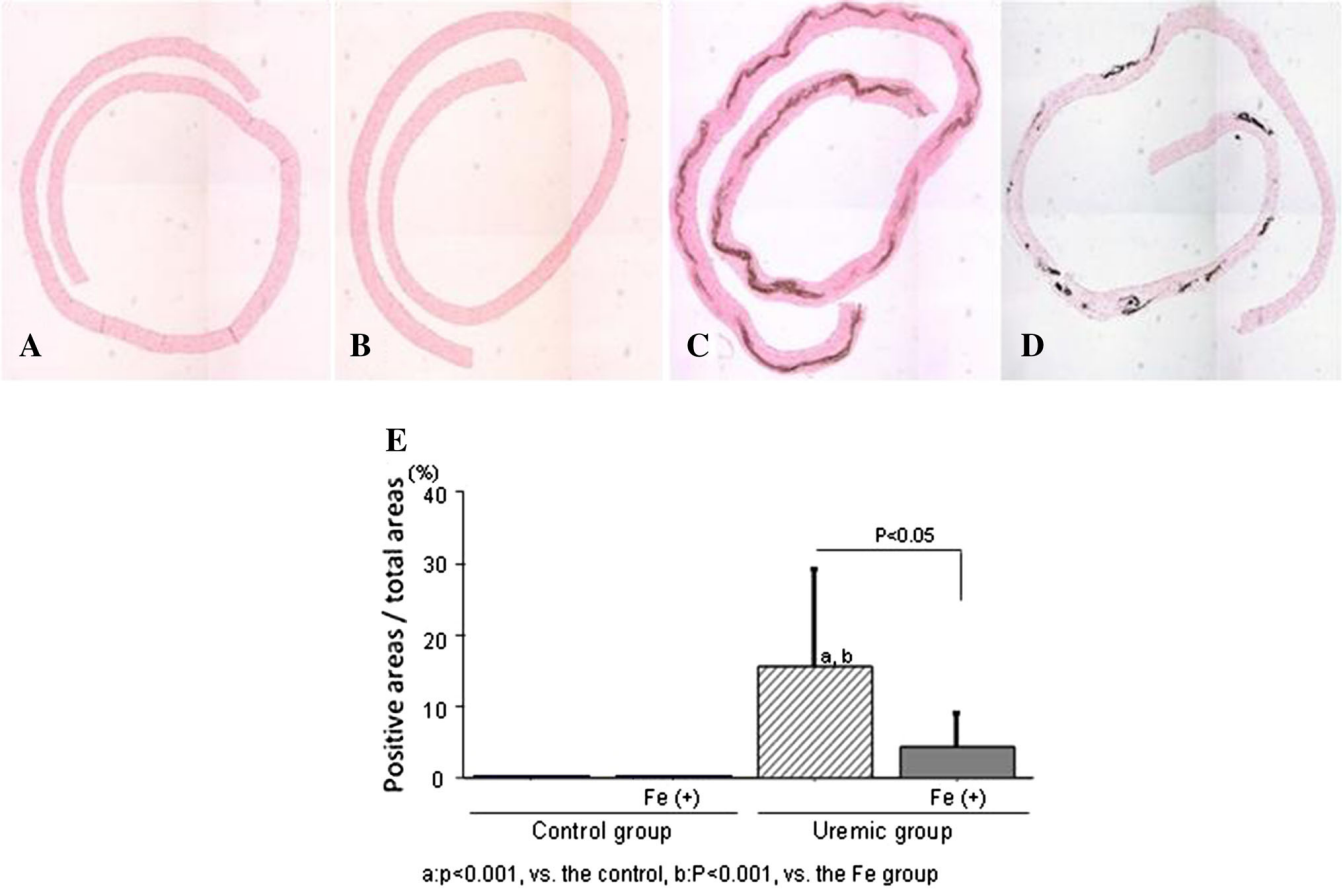
Monocyte adhesion to the endothelium and arterial calcification. Representative von Kossa staining on day 56 in the control group (**a**), the Fe group (**b**), the uremic group (**c**), and the uremic + Fe group (**d**). **e** The area of calcification in the uremic + Fe group was significantly suppressed compared to that in the uremic group (p < 0.05). ^a^p < 0.001 versus the control group, ^b^p < 0.001 versus the Fe group. Data are expressed as mean ± SD in each group

No calcified lesions were observed in the control and Fe only groups (Fig. 2a, b). A calcified band in the thoracic media was observed in the uremic group (Fig. 2c). Fragmented calcification in the thoracic media was observed in the uremic + Fe group (Fig. 2d). The areas of calcification (positive areas in a von Kossa stain) in the uremic rats were significantly larger than those in the control rats (Fig. 2e). The increase of the calcified areas in the uremic rats was remarkably suppressed by Fe administration (Fig. 2F).

### Expression of Pit-1 in the thoracic aorta

**Fig. 3:**
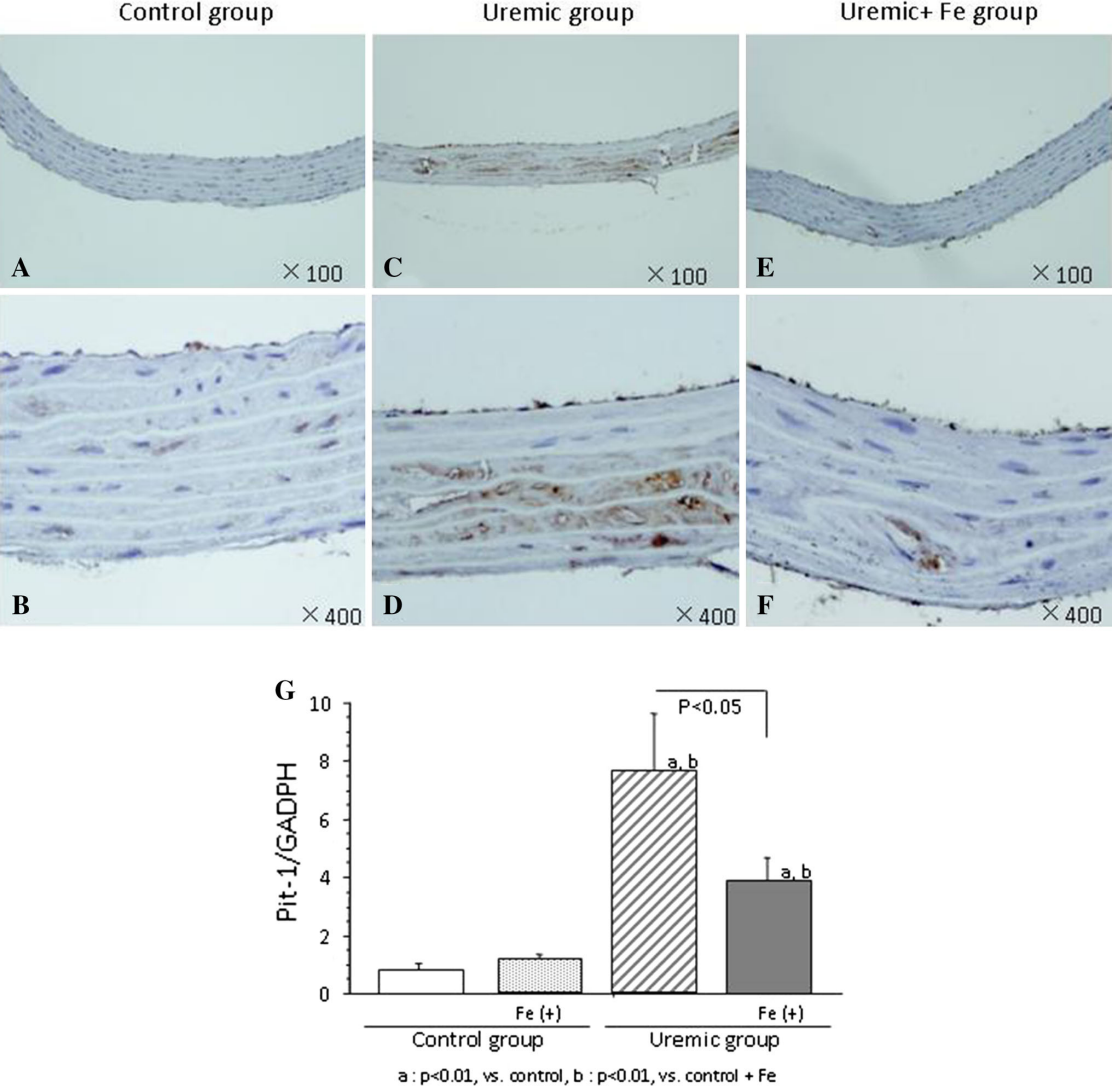
Effect of iron dextran on Pit-1 expression of thoracic aorta. Phosphate transporter (Pit)-1 was not detected in the control and Fe only groups (**a** 100× magnification, **b** 400× magnification). Pit-1 expressing areas were scattered throughout the whole smooth muscle layer (**b** 100× magnification, **c** 400× magnification). Pit-1 expressing areas were found in smooth muscle layer (**e** 100× magnification, **f** 400× magnification). Levels of Pit-1 mRNA in each group are shown **g**. Data are expressed as mean ± SD in each group

Pit-1 was not detected in the control and Fe only groups (Fig. 3a, b). Pit-1 expressing areas were scattered throughout the whole smooth muscle layer (Fig. 3c–f). Dotted Pit-1 expressing areas were found in the smooth muscle layer (Fig. 3e, f). Multi-layered construction in thoracic media was ruptured around Pit-1 expressing areas (Fig. 3d–f). Pit-1 mRNA expression, elevated in the uremic condition, was suppressed by Fe administration (Fig. 3g).

### Expressions of ss-DNA and Runx2

**Fig. 4:**
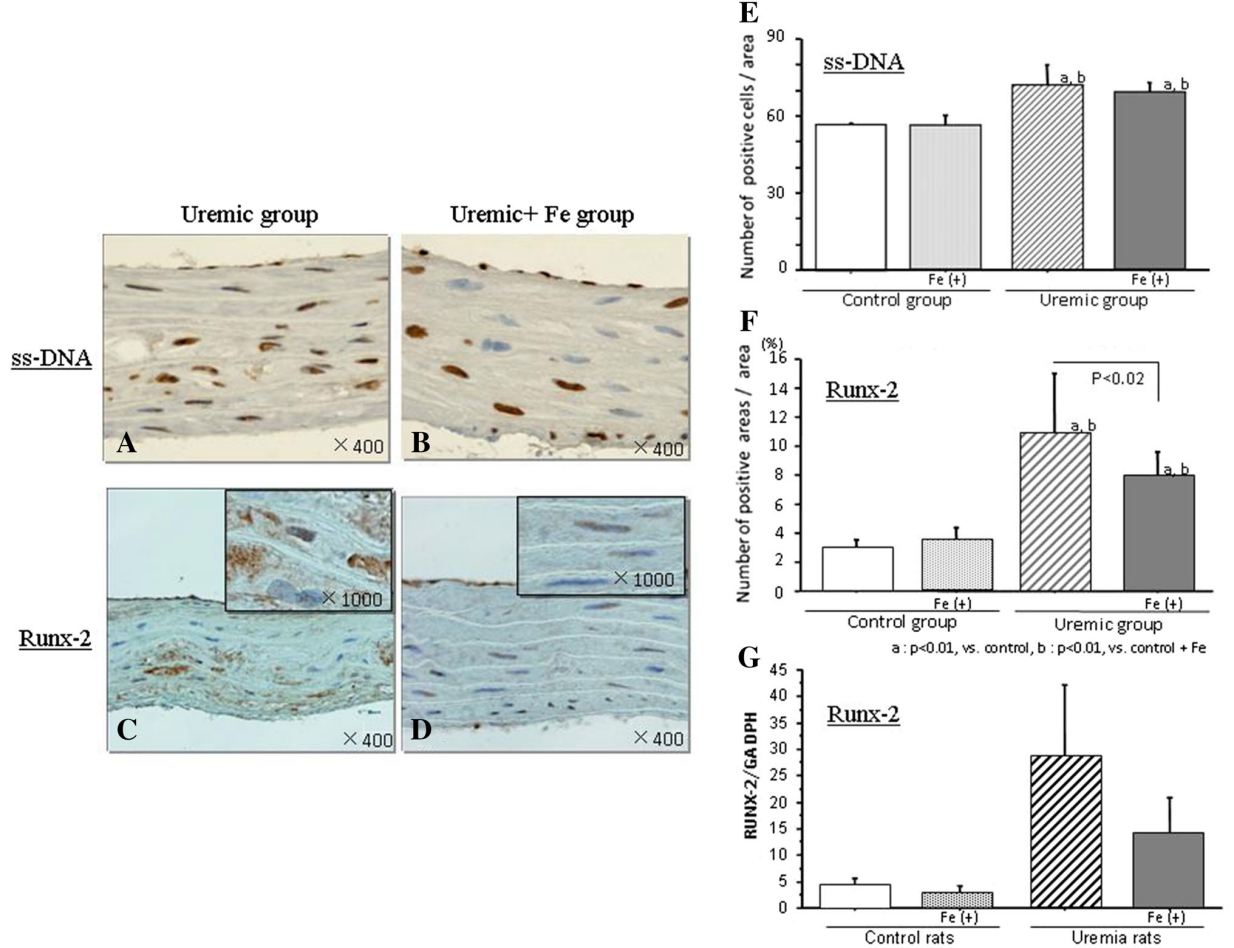
Expressions of apoptotic marker (ss-DNA) and osteo-/chondrogenic conversion marker (Runx2) in the thoracic aorta. Representative immunohistochemical findings of ss-DNA and Runx2 in the uremic rats (**a**–**d)**. Immunohistochemistry for Runx2 **c**, **d** showed a predominantly cytoplasmic distribution pattern in the uremic group compared with the uremic + Fe group. Not Fe but uremic condition increased the number of ss-DNA positive cells (**e**). Increased number of Runx2 positive cells by uremic condition was suppressed by Fe (**f**). Levels of Runx2 mRNA in each group are shown (**g**). Data are expressed as mean ± SD in each group

Many ss-DNA expressing cells, a specific apoptotic marker, were present in the uremic groups (Fig. 4a, b). However, the number of ss-DNA expressing cells in the Fe-treated group was comparable with that in the uremic groups (Fig. 4e). Runx2, an osteo-/chondrogenic conversion marker, was abundantly observed in the vessels of the uremic group. In an immunohistochemical analysis, Runx2 expressing cells were present in the VSMCs (Fig. 4c, d). Expression of Runx2 was observed in cytoplasmic areas in the uremic groups (Fig. 4c, d). Administration of iron dextran (Fe) suppressed increasing Runx2 positive areas and expression of Runx2 mRNA introduced by uremic condition (Fig. 4f, g).

## Discussion

Our examination revealed the following points: (1) uremic condition induced significant vascular calcification in the rats fed a phosphorun enriched diet, (2) Fe suppressed the medial vascular calcification induced by the uremic condition, (3) the suppressive effect of Fe on vascular calcification could be introduced by the inhibition of SMCs transforming to osteoblast/chondrocyte-like cells.

Fe, a strong inducer of oxidative stress, is commonly administered to CKD patients with anemia. A previous study indicated that Fe promotes endothelial injury and dysfunction, as well as monocyte adhesion in cultured endothelial cells [8]. However, Fe administration had no additional effect on these levels in the uremic condition, although marked Fe accumulation in the liver was observed at a sufficient dosage (data not shown). According to these results, the effect of Fe through oxidative stress might be transient on endothelial cells, or there are unknown factors or a cross-relationship surrounding endothelial dysfunction in uremia.

The development of vascular calcification is started by the uptake of phosphate into SMCs through the excessive expression of the phosphate transporter Pit-1 [9]. We confirmed that Fe suppressed the elevated Pit-1 expression induced by uremic condition regardless of serum phosphate level alteration. Since the levels of serum FGF23 decreased in the uremic + Fe group without a decrease of serum phosphate, we suspect that Fe might play a role in the regulation of FGF23 directly or indirectly. The intracellular phosphate uptake induces the development of vascular calcification via two pathways. The first is a matrix vesicle releasing pathway. The rising concentration of intracellular phosphate in SMCs makes hydroxyapatite in the matrix vesicle, and then apoptotic SMCs released in the matrix vesicle become footholds for interstitial calcification depositions. A previous study has reported that SMC apoptosis was induced and accelerated by intracellular phosphate uptake [10]. A uremic condition induced accelerating apoptosis in the vascular walls, but no suppressive effect of Fe was observed in our study. An increase of extracellular phosphate intensifies the expression of Runx2, which has taken the place of myocardin—an essential transcription cofactor located at the genetic locus for SMCs differentiation, and induces the transdifferentiation to osteoblasts [11]. In our study, Fe was able to suppress the SMC transformation into osteoblast-like cells in the uremic condition.

Ferritin is a Fe storage protein that exhibits antioxidant properties and is cytoprotective for the endothelium [6]. Ferritin suppressed calcium deposition and osteoblastic differentiation of VSMCs cultured in a high phosphate medium [12]. Since the levels of ferritin in the Fe-treated groups were significantly elevated, especially in the uremic + Fe rats, ferritin might play a role in the spurring vascular calcification. It is recognized that Fe may have a direct effect on osteoblast function and patients with overt evidence of Fe administration have been shown to have a mineralization defect on bone biopsy [13]. Fe administration ameliorated osteoblastic phenotypic change and function in fetal rat calvaria cultures [14]. This is the first paper to show directly effects of Fe on VSMCs in vivo.

Originally, we hypothesized that Fe administration may enhance vascular calcification in the uremic condition. However, this study has indicated precisely the opposite results. Recently, a new Fe-based phosphate binder is under development [15]. Fe deposition was observed in the vascular medial layer of the arteries in this study (data not shown), which might have a direct physicochemical effect on vascular calcification. Fe itself may play an important role in SMCs as a cytotoxic factor. It is important to further examine the effect of Fe on the endothelial dysfunction in vivo, and the relationship between the endothelial dysfunction and the vascular calcification.

It appears that Fe administration suppresses excessive vascular medial calcification in the uremic condition through the suppression of Pit-1 expression and VSMC transformation into the osteoblast-like cells.
